# Supplementary material for: Design of miniprotein inhibitors targeting complement C9 to block membrane attack complex assembly
Source: Nat Commun. 2026 Mar 12;17:3827. doi: 10.1038/s41467-026-70667-x (PMC13121834; doi:10.1038/s41467-026-70667-x)

Supplementary information

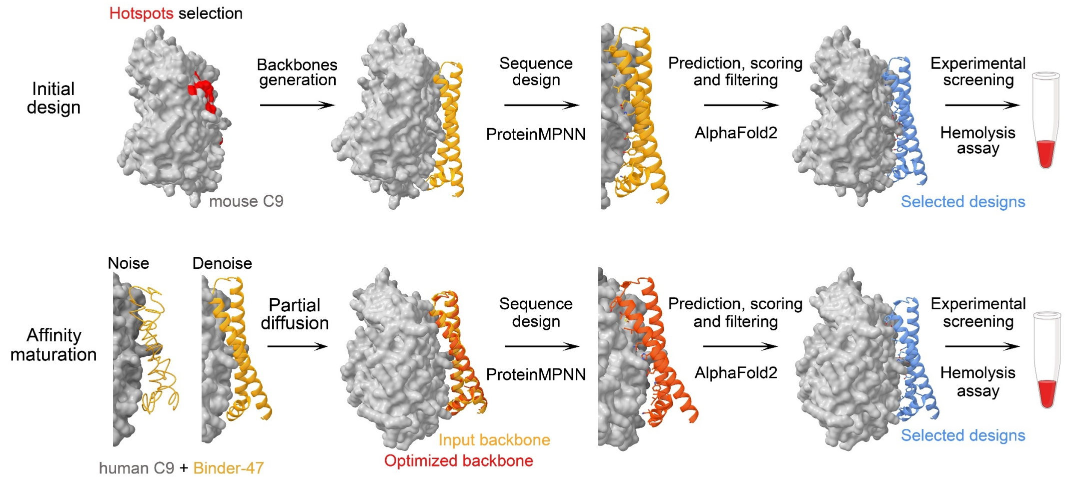

Supplementary Figure 1. Schematic representation of the complement C9 binder design and selection process. The initial design and affinity maturation of the C9 mini-protein binder followed a workflow of hotspot selection, scaffold generation, sequence design, structure prediction, scoring, and screening. Detailed information is provided in the **methods**. In the initial design, we used mouse complement C9. PDB ID: 6CXO. And in the subsequent affinity optimization section, the structure of the human C9 monomer predicted by AlphaFold3 was used.

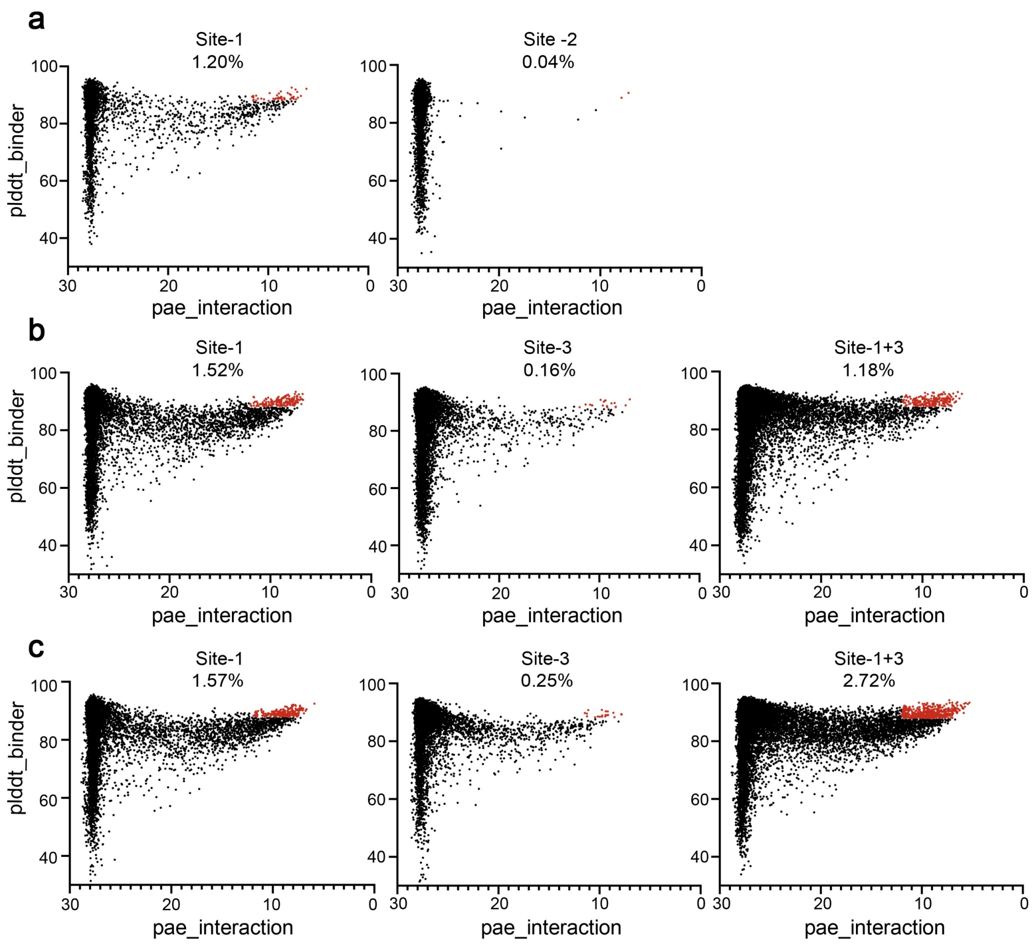

Supplementary Figure 2. AlphaFold2 prediction score for mini-proteins binding with mouse complement C9. The AlphaFold2 predicted scores for the first (**a**), second (**b**) and third (**c**) round calculation results for hotspots Site-1, Site-2 and Site-1+2. Scores that meeting the cut-off pae_interaction < 15 and plddt_binder > 88 are highlighted in red. Source data are provided as a Source Data file.

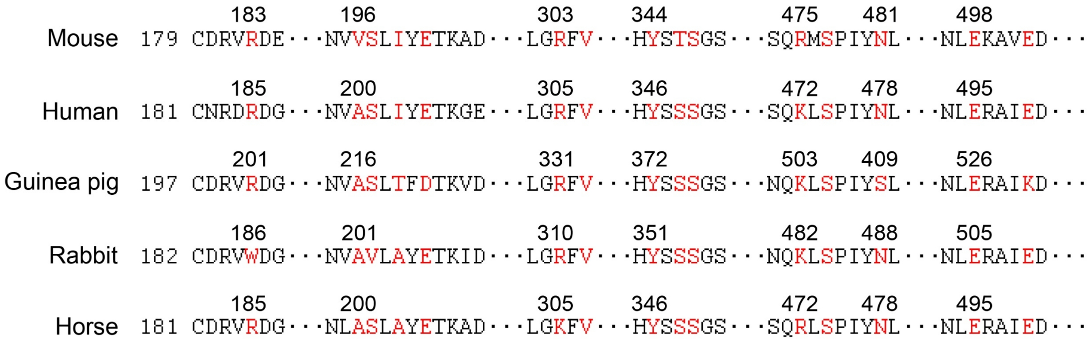

Supplementary Figure 3. Complement C9 amino acid sequences conservation across different species. The binding sites with the mini-protein inhibitors are highlighted in red. The mouse, human, rabbit, horse sequences were obtained from UniProt, ID: P06683, P02748, P48747, P48770. The guinea pig sequence was obtained from NCBI, ID: XM_063258105.1.

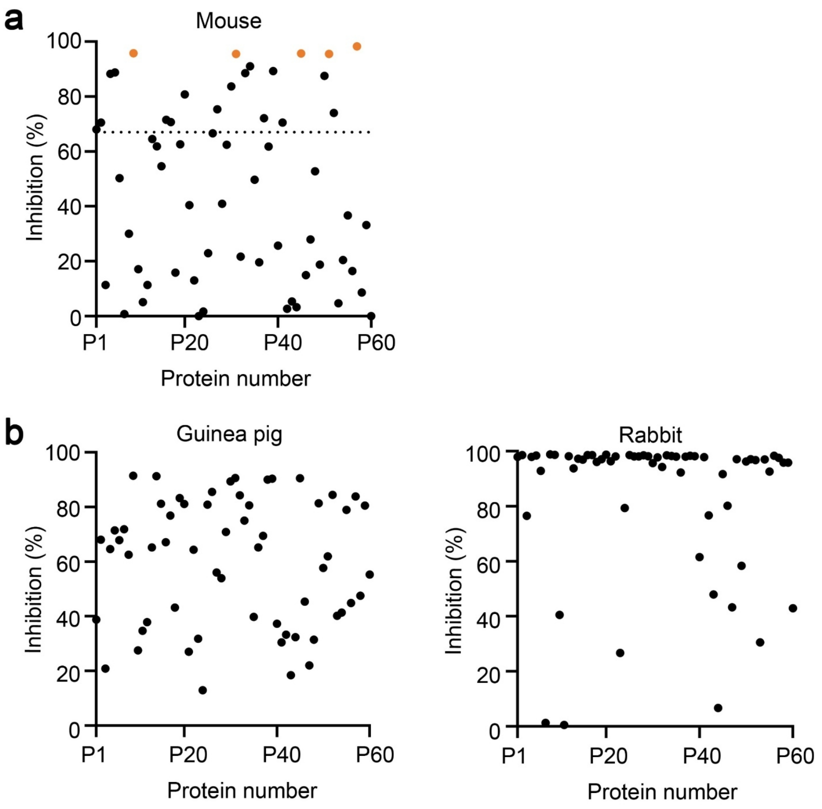

Supplementary Figure 4. In vitro mini-protein binders hemolysis inhibition rates for different species. (a) Inhibition rates of hemolysis inhibition assay mediated by mouse serum. Orange scatter symbols highlight binders that achieve >90 % inhibition of hemolysis. **(b)** Inhibition rates of hemolysis inhibition assay mediated by guinea pig (left) and rabbit (right) serum. The detailed serum concentrations and mini-protein inhibitors concentrations are mentioned in **Methods** and **Supplementary Table 1**. And source data are provided as a Source Data file.

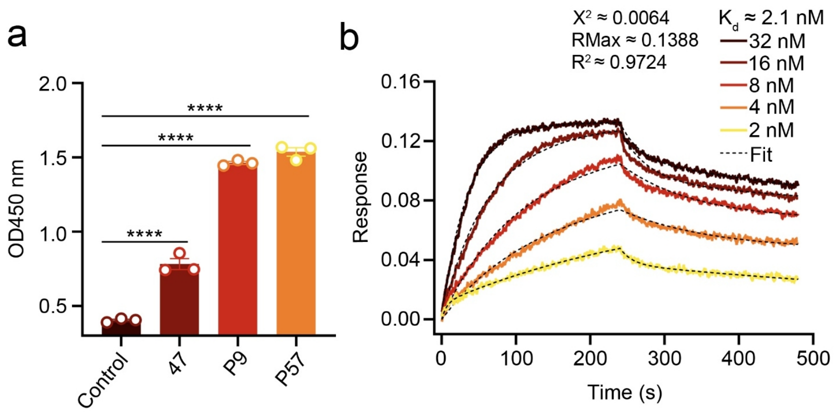

Supplementary Figure 5. ELISA and BLI experiments for mini-protein inhibitors. (a) Indirect ELISA of Binder-47, P9 and P57. **(b)** BLI characterization of P57 mini-protein binder and human complement C9. Graphs in **a** show mean ± SEM of n = 3 biologically independent samples. Statistical significance was determined by ordinary one-way ANOVA followed by two-sided Dunnett's multiple comparisons test. F values, degrees of freedom (df), and adjusted P values are provided in the **Supplementary Table 4**. ****$P < 0.0001$. Source data are provided as a Source Data file.

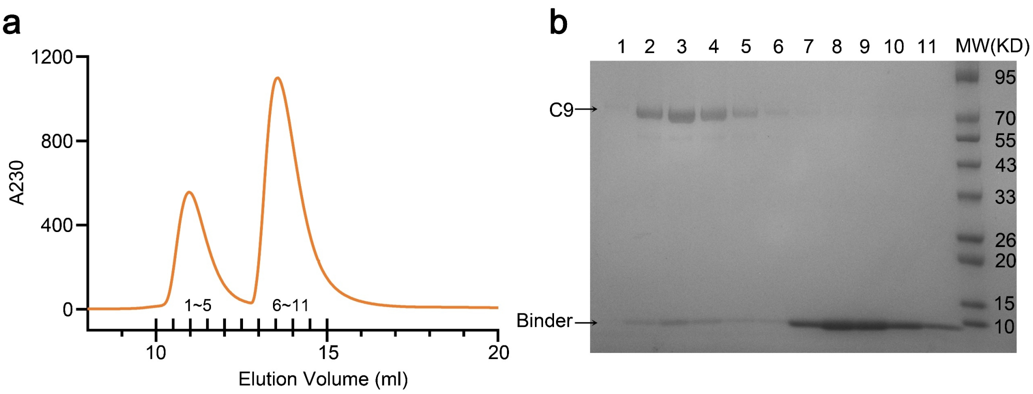

Supplementary Figure 6. Complex formation between mini-protein binder P57 and complement C9. (a) SEC profile of recombinant mouse C9 in complex with the P57 mini-protein binder prior to crystallization. **(b)** The SDS-PAGE analysis corresponding to elution tube of **a**. Source data and uncropped gel image are provided as a Source Data file. And uncropped gel image also available below this file.

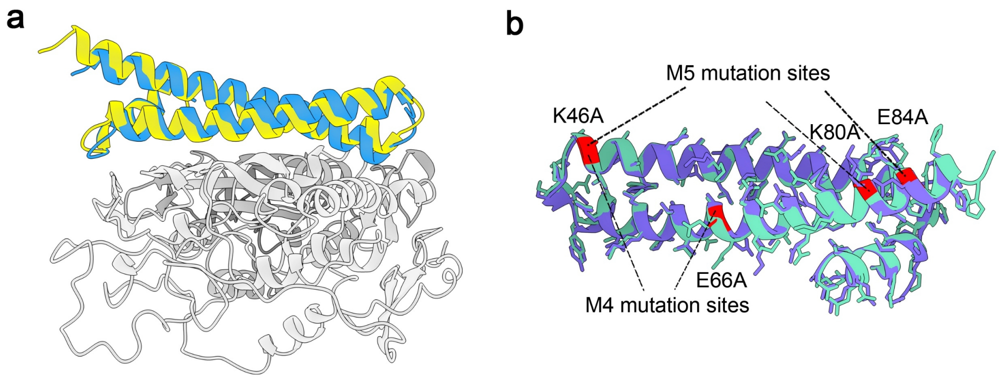

Supplementary Figure 7. Crystal structure of the complement C9 mini-protein inhibitors and surface alanine mutations for crystallization. (a) Superimposition of the P57-M5 crystal structure and the computational design model of the C9–P57 complex. Blue, computational design model of P57. Yellow, the P57-M5 crystal structure. Gray, the human complement C9 structure. **(b)** Superimposition of the P57-M4 and P57-M5 crystal structure. Residues selected for mutation to alanine are highlighted in red. Purple, P57-M4. Cyan, P57-M5. The detailed amino acid sequences are presented in **Supplementary Table 2**.

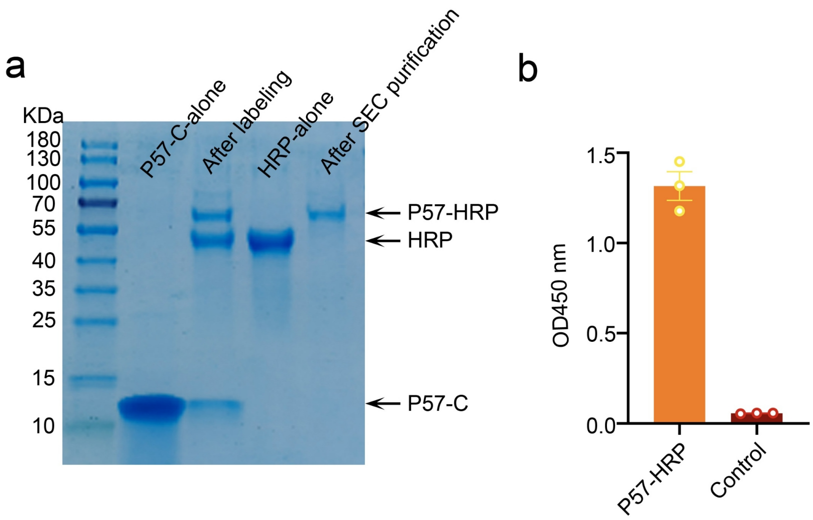

Supplementary Figure 8. The characterization of HRP labeled mini-protein binder P57-HRP. (a) SDS-PAGE analysis of the SEC purified P57-HRP. **(b)** Direct ELISA was employed to quantify the interaction between human complement C9 and P57-HRP. Graphs in **b** show mean ± SEM of n = 3 biologically independent samples. Uncropped gel image and source data are provided as a Source Data file. And uncropped gel image also available below this file.

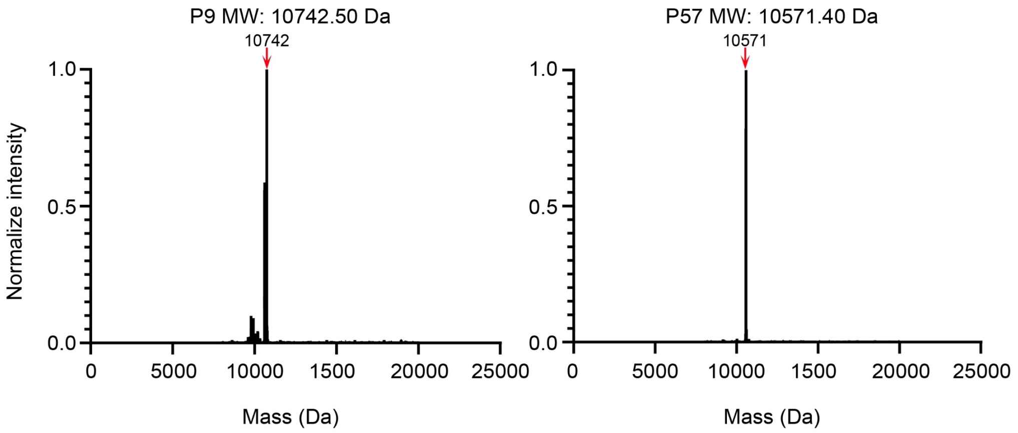

Supplementary Figure 9. Mass spectrometry analysis of P9 and P57. The theoretical molecular mass of each protein is indicated at the top of the corresponding panel, and the observed peak for the intact protein is highlighted with a red arrow. The amino acid sequences are listed in **Supplementary Table 1**. Source data are provided as a Source Data file.

a

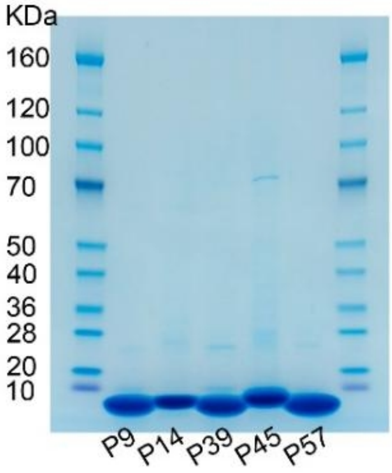

b

Protein number	Yield (mg/L)
P9	49.98
P14	24.58
P39	79.4
P45	8.75
P57	126.85

Supplementary Figure 10. Yields of human complement C9 mini-protein inhibitors. (a) After Ni-NTA affinity purification, 5 μg of each mini-protein binder was run through SDS-PAGE. **(b)** Mini-protein binders yield from 1 L expression culture. Protein concentrations were determined using a multimode microplate reader by measuring the absorbance at 280 nm. Uncropped gel image is provided as a Source Data file, and it is also available below this file.

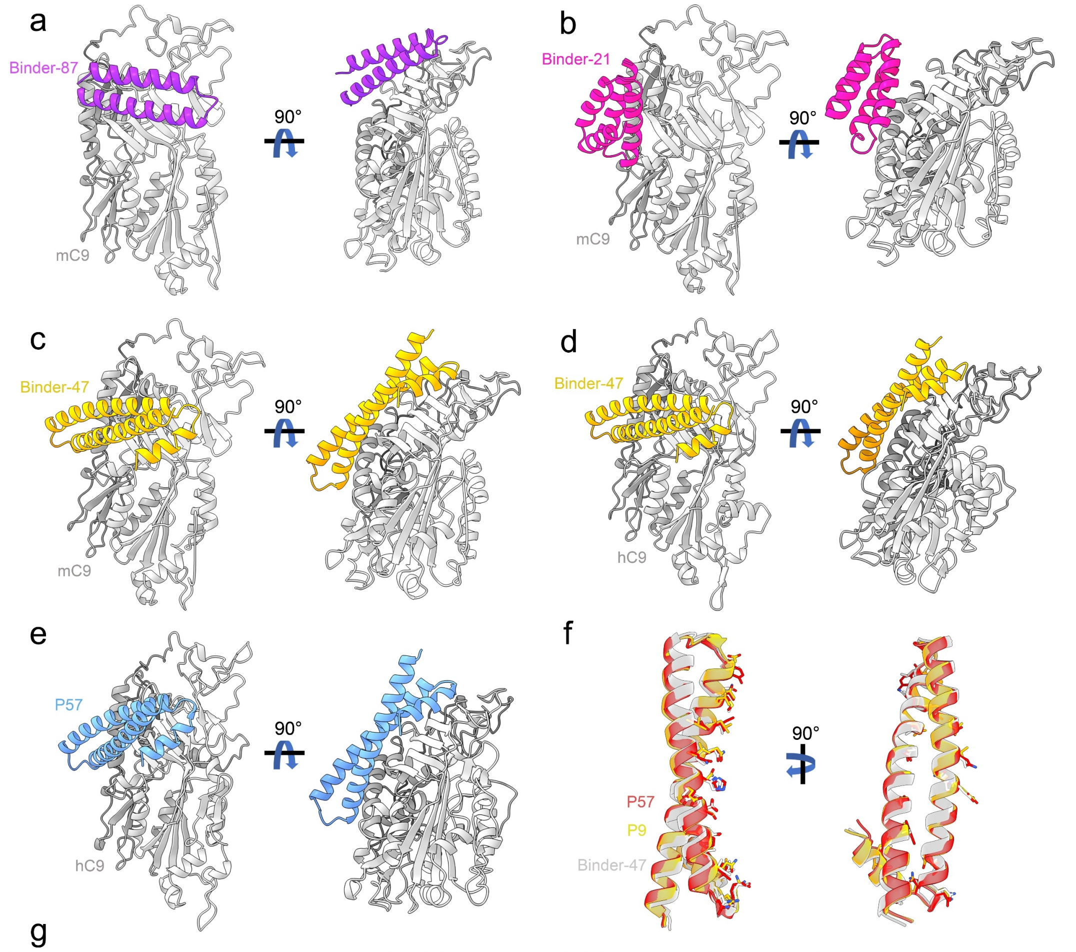

g

	pae_binder	pae_interaction	pae_target	plddt_binder	plddt_target	plddt_total	pTM	ipTM
binder-87 (AF2)	2.649	6.490	4.305	90.779	95.906	95.396	-	-
binder-21 (AF2)	2.254	6.959	4.392	91.025	95.657	95.038	-	-
binder-47 (AF2)	3.022	6.115	4.336	92.327	95.688	95.165	-	-
P57-hC9 (AF2)	3.219	6.088	4.984	92.420	94.542	94.233	-	-
binder-47-hC9 (AF3)	-	-	-	-	-	-	0.85	0.93
binder-47 (AF2)	-	-	-	92.327	-	-	-	-
P9 (AF2)	-	-	-	92.692	-	-	-	-
P57 (AF2)	-	-	-	92.420	-	-	-	-

Supplementary Figure 11. AlphaFold models from two viewing angles and the relevant scores. (a) The prediction model of Binder-87 (purple) with mouse complement C9 (mC9, gray) shown in **Figure 1c** left. **(b)** The prediction model of Binder-21 (pink) with mouse complement C9 (mC9, gray) shown in **Figure 1c** middle. **(c)** The prediction model of Binder-47 (orange) with mouse complement C9 (mC9, gray) shown in **Figure 1c** right. **(d)** The prediction model of Binder-47 (orange) and human complement C9 (hC9, gray) shown in **Figure 2a**. **(e)** The prediction model of P57 (blue) with human complement C9 (hC9, gray) shown in **Figure 4a** and **Supplementary Figure 7a**. **(f)** Superimposition of the predicted models of Binder-47

(silver), P9 (orange) and P57 (red) shown in **Figure 3c**. Except for **d,** which was predicted using AlphaFold3 (AF3), all other structures were predicted using AlphaFold2 (AF2). **(g)** The scores related to the prediction models shown in **a**, **b**, **c**, **d**, **e**, and **f**. All tested models are available on zenodo.org (accession code: 18530064).

Supplementary Table 1 Alanine mutation sequences of P9 and P57 anti C9 mini-protein binders for crystal screening.

	Sequence (bold represent for native sequence)	Crystal	Structure solved
P9-native	**MSAEELAALLLQNENGVEQAKLAVESLEENYKLLDELAKEYAAKAAADPENDGYKTLAKLYAKQAEETKKELKEYTEALKLLEERRHHHHHH**	√	
P9-M1	MSAEELAALLLQNENGVEQAKLAVESLEENYKLLDELAKEYAAKAAADPENDGYKTLAKLYAKQAEETKKELKEYTaALaLLEaRRHHHHHH	√	
P9-M2	MSAEELAALLLQNENGVEQAKLAVESLEENYKLLDELAaEYAAKAAADPENDGYKTLAKLYAKQAEETaKELaEYTEALKLLEERRHHHHHH	√	
P9-M3	MSAEELAALLLQNENGVEQAKLAVESLEENYKLLDELAKEYAAKAAADPENDGYKTLAKLYAKQAEETaKELaEYTEALKLLEaRRHHHHHH	√	
P57-native	**MNAEELVKALSKQDNPVEIAREALAALQDHLDQLKADAEKWAAKVKADPSNYGAQTMLKIATTQAEELQKEAEEWEKALKALEEAKHHHHHH**	√	
P57-M4	MNAEELVKALSKQDNPVEIAREALAALQDHLDQLKADAEKWAAKVaADPSNYGAQTMLKIATTQAaELQKEAEEWEKALKALEEAKHHHHHH	√	√
P57-M5	MNAEELVKALSKQDNPVEIAREALAALQDHLDQLKADAEKWAAKVaADPSNYGAQTMLKIATTQAEELQKEAEEWEKALaALEaAKHHHHHH	√	√
P57-M6	MNAEELVKALSKQDNPVEIAREALAALQDHLDQLKADAEKWAAKVKADPSNYGAQTMLKIATTQAEELQKEAEEWaKALaALEaAKHHHHHH	√	

Supplementary Table 2 Data collection and refinement statistics.

	P57-M4	P57-M5
**Data collection**		
Space group	P 2_1 2_1 2_1	P 2_1 2_1 2 $_1$
Cell dimensions		
a, *b*, *c* (Å)	42.4 45.0 56.7	42.8 45.9 57.0
a, b, g (°)	90, 90, 90	90, 90, 90
Resolution (Å)	27.13-1.40 (1.45-1.4)	28.53-1.80 (1.86-1.80)
R_{merge}	0.05807 (0.3315)	0.03455 (0.07905)
I / s*I*	19.75 (1.90)	56.27 (7.06)
Completeness (%)	60.7 (45.5)	99.51 (97.03)
Redundancy	2.4 (2.2)	4.2 (2.6)
**Refinement**		
Resolution (Å)	27.13-1.46 (1.53-1.46)	28.53-1.8 (1.88-1.80)
No. reflections		
Working set	12960 (963)	10873 (1046)
Test set	1304 (104)	1078 (106)
Final R_{work}	0.1950 (0.1779)	0.2030 (0.2288)
Final R_{free}	0.2300 (0.2234)	0.2200 (0.3135)
No. atoms		
Protein	691	697
Ligand/ion	0	0
Water	110	121
Average B-factors	17.42	25.86
Protein	15.3	24.06
Water	30.76	36.24
R.m.s. deviations		
Bond lengths (Å)	0.008	0.008
Bond angles (°)	1.12	1.12
Ramachandran plot		
Favored (%)	100	98.86
Additionally (%)	0	1.14
Outliers (%)	0	0
PDB ID	9X1W	9X1X

Each crystal structure solved by analyzing data obtained from diffraction of one crystal.

*Values in parentheses are for highest-resolution shell.

Uncropped gel image of **Supplementary Figure 6b.**

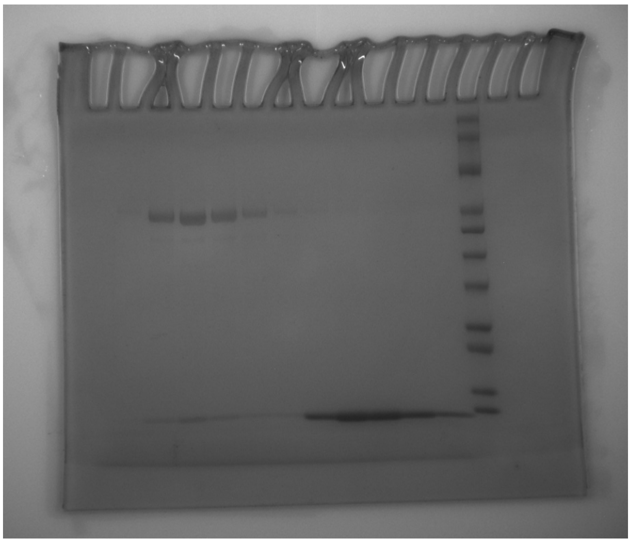

Uncropped gel image of **Supplementary Figure 8a.**

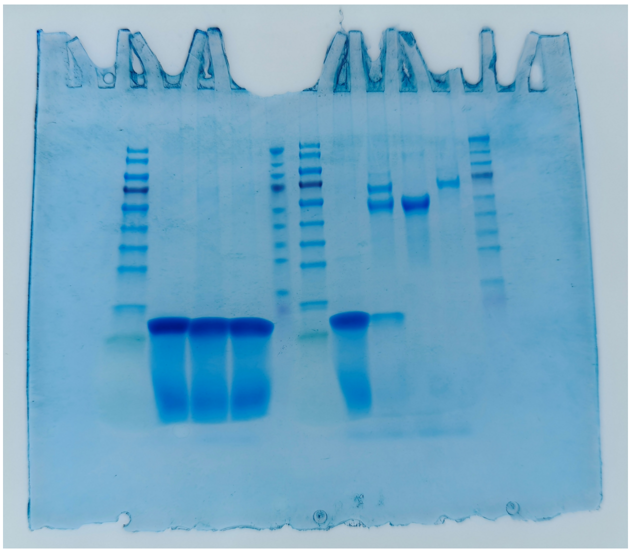

Uncropped gel image of **Supplementary Figure 10a.**

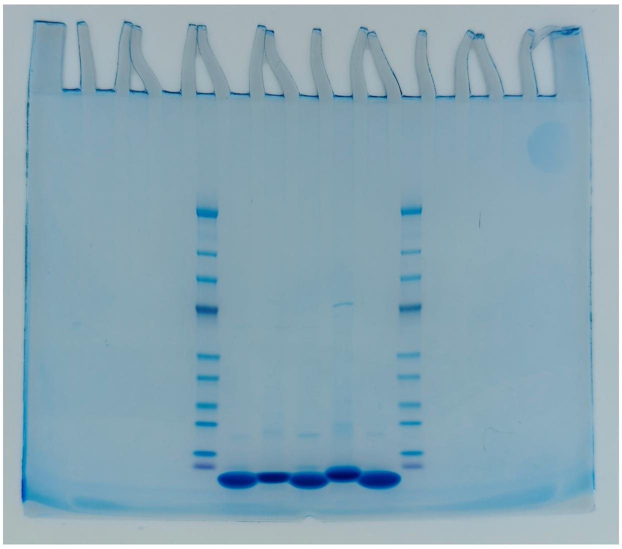

Supplement: Supplementary file 1 — Supplementary Information [file 41467_2026_70667_MOESM1_ESM.pdf]
