# Supplementary material for: Design of miniprotein inhibitors targeting complement C9 to block membrane attack complex assembly
Source: Nat Commun. 2026 Mar 12;17:3827. doi: 10.1038/s41467-026-70667-x (PMC13121834; doi:10.1038/s41467-026-70667-x)
Supplement: Supplementary file 2 — Description of Additional Supplementary Files [file 41467_2026_70667_MOESM2_ESM.pdf]

Description of Additional Supplementary Files

Supplementary Data 1. The sequences of each anti C9 mini-protein binders and their inhibition rates (%) against different sera.

Supplementary Data 2. Significance analysis of data. ns (non-significant); * $P \leq 0.05$; ** $P \leq 0.01$; *** $P \leq 0.001$; **** $P \leq 0.0001$.
