# Supplementary material for: Design of miniprotein inhibitors targeting complement C9 to block membrane attack complex assembly
Source: Nat Commun. 2026 Mar 12;17:3827. doi: 10.1038/s41467-026-70667-x (PMC13121834; doi:10.1038/s41467-026-70667-x)
Supplement: Supplementary file 5 — Reporting Summary [file 41467_2026_70667_MOESM5_ESM.pdf]

nature portfolio

Corresponding author(s): Bowen Yu

Last updated by author(s): Feb 11, 2026

Reporting Summary

Nature Portfolio wishes to improve the reproducibility of the work that we publish. This form provides structure for consistency and transparency in reporting. For further information on Nature Portfolio policies, see our Editorial Policies and the Editorial Policy Checklist.

Statistics

For all statistical analyses, confirm that the following items are present in the figure legend, table legend, main text, or Methods section.

n/a	Confirmed	
☐	☒	The exact sample size (*n*) for each experimental group/condition, given as a discrete number and unit of measurement
☐	☒	A statement on whether measurements were taken from distinct samples or whether the same sample was measured repeatedly
☐	☒	The statistical test(s) used AND whether they are one- or two-sided<br>*Only common tests should be described solely by name; describe more complex techniques in the Methods section.*
☐	☒	A description of all covariates tested
☐	☒	A description of any assumptions or corrections, such as tests of normality and adjustment for multiple comparisons
☐	☒	A full description of the statistical parameters including central tendency (e.g. means) or other basic estimates (e.g. regression coefficient) AND variation (e.g. standard deviation) or associated estimates of uncertainty (e.g. confidence intervals)
☐	☒	For null hypothesis testing, the test statistic (e.g. *F*, *t*, *r*) with confidence intervals, effect sizes, degrees of freedom and *P* value noted<br>*Give P values as exact values whenever suitable.*
☒	☐	For Bayesian analysis, information on the choice of priors and Markov chain Monte Carlo settings
☒	☐	For hierarchical and complex designs, identification of the appropriate level for tests and full reporting of outcomes
☒	☐	Estimates of effect sizes (e.g. Cohen's *d*, Pearson's *r*), indicating how they were calculated

Our web collection on statistics for biologists contains articles on many of the points above.

Software and code

Policy information about availability of computer code

Data collection	Protein design code: https://github.com/RosettaCommons/RFdiffusion; https://github.com/nrbennet/dl_binder_design. Absorbance measurement: SPARKCONTROL 4.1. X-ray crystallography: CrysAlisPro. BLI: Fortebio data acquisition 12.0.1.8. SEC-MALS: ASTRA™ 8.22.115 and HPLC CONNECT™ 4.0.3.41. Mass spectrometry: Masslynx 4.1. SEC: CDsystem 3.2.8 software. CD: ProData Viewer application 4.7.0.194.
Data analysis	X-ray crystallography: CrysAlisPro 171.43.136a(data integration/ scaling), Phenix v.1.20.1-4487 (molecular replacement, model building refinement), WinCoot v.0.9.8.1 (visualization, real time refinement), Pymol v.3.0.4 (molecular graphics), ChimeraX v.1.8 (molecular visualization). Mass spectrometry: Masslynx 4.1. SEC: CDsystem 3.2.8. BLI: Fortebio data analysis 12.0.1.2. Multimode microplate reader v4.1, Graphpad Prism 8.0.2.

For manuscripts utilizing custom algorithms or software that are central to the research but not yet described in published literature, software must be made available to editors and reviewers. We strongly encourage code deposition in a community repository (e.g. GitHub). See the Nature Portfolio guidelines for submitting code & software for further information.

Data

Policy information about availability of data

All manuscripts must include a data availability statement. This statement should provide the following information, where applicable:

- Accession codes, unique identifiers, or web links for publicly available datasets
- A description of any restrictions on data availability
- For clinical datasets or third party data, please ensure that the statement adheres to our policy

Coordinates and structure files have been deposited to the Protein Data Bank with accession codes 9X1W (P57-M4) [https://doi.org/10.2210/pdb9X1W/pdb], 9X1X (P57-M5) [https://doi.org/10.2210/pdb9X1X/pdb]. Full raw data including all tested models are available on zenodo.org (accession code: 18530064) [https://doi.org/10.5281/zenodo.18530064]. The final sequences of the designed inhibitors optimized by partial diffusion are provided in Supplementary Table 1. Source data is available with this paper as a Source Data file.

Research involving human participants, their data, or biological material

Policy information about studies with human participants or human data. See also policy information about sex, gender (identity/presentation), and sexual orientation and race, ethnicity and racism.

Reporting on sex and gender	To exclude the influence of sex on complement activity, only males were recruited in the experiments.
Reporting on race, ethnicity, or other socially relevant groupings	n/a
Population characteristics	n/a
Recruitment	Ten healthy male volunteers aged 20–35 years, with no history of infectious or autoimmune diseases, were recruited for blood collection.
Ethics oversight	The studies involving human samples were approved by Medical Research Ethics Committee of Shandong Second Medical University (NO.2025YX196). The studies were conducted in accordance with the local legislation and institutional requirements. The participants provided their written informed consent to participate in this study.

Note that full information on the approval of the study protocol must also be provided in the manuscript.

Field-specific reporting

Please select the one below that is the best fit for your research. If you are not sure, read the appropriate sections before making your selection.

[x] Life sciences [ ] Behavioural & social sciences [ ] Ecological, evolutionary & environmental sciences

For a reference copy of the document with all sections, see nature.com/documents/nr-reporting-summary-flat.pdf

Life sciences study design

All studies must disclose on these points even when the disclosure is negative.

Sample size	For protein design expression and in vitro hemolysis inhibition test, 163 designs including 103 for initial screening and 60 for affinity maturation. Designs were chosen according to top scoring in silico prediction metrics. The number of designed proteins for experimental testing was determined based on the feasibility of the experiment and the reported success rate of RFdiffusion binder design. The relatively higher number of tests is to ensure get the high-affinity miniprotein binders. For other experiments, number of replication samples are listed in figure captions and methods sections.
Data exclusions	There is no data exclusion in this study.
Replication	Except for the two in vitro hemolysis inhibition tests screens , all other hemolysis assays were repeated at least three times using biologically independent samples. For the first animal experiment in which serum was incubated with the inhibitor, n ≥ 5 biologically independent samples were used. For the second animal experiment that assessed inhibitor activity during ongoing hemolysis, n ≥ 3 biologically independent samples were used. Other experiments including BLI and CD were repeated at least three times with all success result according to the experimental procedure we described. The data analysis was also highly reproducible.
Randomization	Sheep red blood cells were randomly allocated into each reaction.
Blinding	To ensure experimental controllability, researchers were not blinded to different samples in the hemolysis inhibition assays.

Reporting for specific materials, systems and methods

We require information from authors about some types of materials, experimental systems and methods used in many studies. Here, indicate whether each material, system or method listed is relevant to your study. If you are not sure if a list item applies to your research, read the appropriate section before selecting a response.

Materials & experimental systems

n/a	Involved in the study
☐	☒ Antibodies
☒	☐ Eukaryotic cell lines
☒	☐ Palaeontology and archaeology
☐	☒ Animals and other organisms
☒	☐ Clinical data
☒	☐ Dual use research of concern
☒	☐ Plants

Methods

n/a	Involved in the study
☒	☐ ChIP-seq
☒	☐ Flow cytometry
☒	☐ MRI-based neuroimaging

Antibodies

Antibodies used	Rabbit anti-sheep erythrocyte antibody (Beijing Bersee Biotechnology, BM351Y), eculizumab (AstraZeneca), C9 antibody (E-3; Santa Cruz Biotechnology, sc-390000), X197 antibody (Hycultbiotech, HM2111), An rabbit anti-6*His HRP-conjugated antibody (Proteintech, HRP-84814).
Validation	The rabbit anti-sheep erythrocyte antibody (Beijing Bersee Biotechnology, BM351Y) was verified to sensitize sheep erythrocytes (Zhengzhou Pingrui Biotechnology Co., Ltd., ZPRMYHXB-100/4) at a dilution of 1:400 when using mouse serum in an vitro hemolysis inhibition assay. In addition, other antibodies include the complement C5 inhibitor eculizumab (AstraZeneca), the C9 antibody (E-3; Santa Cruz Biotechnology, sc-390000), and the X197 antibody (Hycultbiotech, HM2111), which were used to compare their biological functions with the mini-protein inhibitors we designed, to determine whether they could effectively inhibit the occurrence of complement-mediated hemolysis. The rabbit anti-6*His HRP-conjugated antibody (Proteintech, HRP-84814) was validated for detection of 6*His tagged proteins in ELISA.

Animals and other research organisms

Policy information about studies involving animals; ARRIVE guidelines recommended for reporting animal research, and Sex and Gender in Research

Laboratory animals	BALB/c mouse (8-week-old), guinea pig (8-week-old) and rabbit (8-week-old).
Wild animals	The study did not involve wild animals.
Reporting on sex	To exclude the influence of sex on complement activity, only male animals were used in the experiments.
Field-collected samples	The study did not involve wild animals.
Ethics oversight	This study was approved by the Ethics Review Board of Shandong Second Medical University. The animal experiments were conducted in strict adherence to the guidelines and regulations set forth by the Laboratory Monitoring Committee of Shandong Province, China, and were approved by the Experimental Animal Ethics Committee of Shandong Second Medical University (NO.2025SDL798).

Note that full information on the approval of the study protocol must also be provided in the manuscript.

Plants

Seed stocks	*Report on the source of all seed stocks or other plant material used. If applicable, state the seed stock centre and catalogue number. If plant specimens were collected from the field, describe the collection location, date and sampling procedures.*
Novel plant genotypes	*Describe the methods by which all novel plant genotypes were produced. This includes those generated by transgenic approaches, gene editing, chemical/radiation-based mutagenesis and hybridization. For transgenic lines, describe the transformation method, the number of independent lines analyzed and the generation upon which experiments were performed. For gene-edited lines, describe the editor used, the endogenous sequence targeted for editing, the targeting guide RNA sequence (if applicable) and how the editor was applied.*
Authentication	*Describe any authentication procedures for each seed stock used or novel genotype generated. Describe any experiments used to assess the effect of a mutation and, where applicable, how potential secondary effects (e.g. second site T-DNA insertions, mosiacism, off-target gene editing) were examined.*
